# A novel isoflavone, ME-344, targets the cytoskeleton in acute myeloid leukemia

**DOI:** 10.18632/oncotarget.10446

**Published:** 2016-07-06

**Authors:** Danny V. Jeyaraju, Rose Hurren, Xiaoming Wang, Neil MacLean, Marcela Gronda, Aisha Shamas-Din, Mark D. Minden, Guri Giaever, Aaron D. Schimmer

**Affiliations:** ^1^ Princess Margaret Cancer Centre, University Health Network, Toronto, ON, Canada; ^2^ Department of Pharmaceutical Sciences, University of British Columbia, Vancouver, BC, Canada

**Keywords:** tubulin, leukemia, synergy, mitochondria, reactive oxygen species

## Abstract

The isoflavone ME-344 is a potent anti-cancer agent with preclinical and clinical efficacy in solid tumors. Yet, the mechanism of action of ME-344 has not been fully defined and the preclinical efficacy in leukemia has not been established. Therefore, we investigated the anti-leukemic properties and mechanism of action of ME-344. In a panel of 7 leukemia cell lines, ME-344 was cytotoxic with an IC_50_ in the range of 70–260 nM. In addition, ME-344 was cytotoxic to primary AML patient samples over normal hematopoietic cells. In an OCI-AML2 xenograft model, ME-344 reduced tumor growth by up to 95% of control without evidence of toxicity. Mechanistically, ME-344 increased mitochondrial ROS generation in leukemic cells. However, antioxidant treatment did not rescue cell death, suggesting that ME-344 had additional targets beyond the mitochondria. We demonstrated that ME-344 inhibited tubulin polymerization by interacting with tubulin near the colchicine-binding site. Furthermore, inhibition of tubulin polymerization was functionally important for ME-344 induced death. Finally, we showed that ME-344 synergizes with vinblastine in leukemia cells. Thus, our study demonstrates that ME-344 displays preclinical efficacy in leukemia through a mechanism at least partly related to targeting tubulin polymerization.

## INTRODUCTION

Acute myeloid leukemia (AML) is the most common form of acute leukemia in adults, and its incidence increases with age [1]. Adults of more than 60 years of age have a less favorable prognosis than younger individuals due to toxicity and poor response rates of standard induction chemotherapy. In addition, relapsed and refractory AML is a highly aggressive and resistant disease with minimal response to chemotherapy [1, 2]. Therefore, there is a need to develop new and improved therapies with low toxicity for the treatment of AML.

Isoflavone derivatives are potential novel therapies for the treatment of malignancies. Isoflavones are related to naturally occurring flavonoids, a class of secondary metabolites with phytoesterogen – plant-derived compounds with estrogenic properties. Some of the well-known isoflavones such as genistein, daidzein and phenoxodiol exhibit anti-cancer effects against various human cancers with high specificity [3–7]. Phenoxodial is the synthetic derivative of naturally occurring genistein that induces cell death in chemoresistant ovarian cancer cells through the mitochondrial apoptotic pathway via caspase-2 mediated cleavage of Bid and proteasomal degradation of XIAP [6–8].

NV-128 is a second generation isoflavone derivative similar to phenoxodiol and induces caspase-independent cell death in epithelial ovarian cancer stem cells in part by targeting the mitochondria. NV-128 inhibits mTOR, causing mitochondrial depolarization and nuclear translocation of EndoG that kills cancer cells by cleaving nuclear DNA and condensing chromatin [9, 10]. NV-128 also activates mitochondrial MAP/ERK kinase pathway and causes a loss of mitochondrial potential by upregulating Bax [9]. In addition, NV-128 facilitates the degradation of mitochondrial oxidative phosphorylation complex IV subunits Cox-I and Cox-IV by an unknown mechanism, and produces mitochondrial reactive oxygen species (ROS) leading to death of malignant cells [9, 10].

ME-344 is the active demethylated metabolite of NV-128 that is more cytotoxic to cancer cells than NV-128 in preclinical studies [9, 10]. While the specific cellular target of ME-344 is unknown, it induces cell death by interfering with mitochondrial oxidative phosphorylation by reducing the activity of complex I in HEK293T cells. This results in a reduction of mitochondrial oxygen consumption, a loss of mitochondrial membrane potential, and activation of the mitochondrial cell death signaling pathways [11]. In a recently reported phase I clinical trial in patients with refractory solid tumors, ME-344 was well tolerated and produced a partial clinical response in one patient and stabilized the disease in four others [12]. Currently, a phase 1b clinical trial of ME-344 in combination with the topoisomerase inhibitor, topotecan, is underway in patients with solid small cell lung and ovarian cancers [13].

Despite the preclinical and clinical efficacy of ME-344 in solid tumors, its activity in leukemia has not been established. In addition, the cellular target of ME-344 is unknown and the mechanism of action has not been fully defined. In this study, we have investigated the preclinical efficacy of ME-344 in leukemia and defined a new mechanism of action targeting tubulin polymerization, which is distinct from vinca alkaloids.

## RESULTS

### ME-344 is cytotoxic to leukemic cells *in vitro*

**Figure 1:**
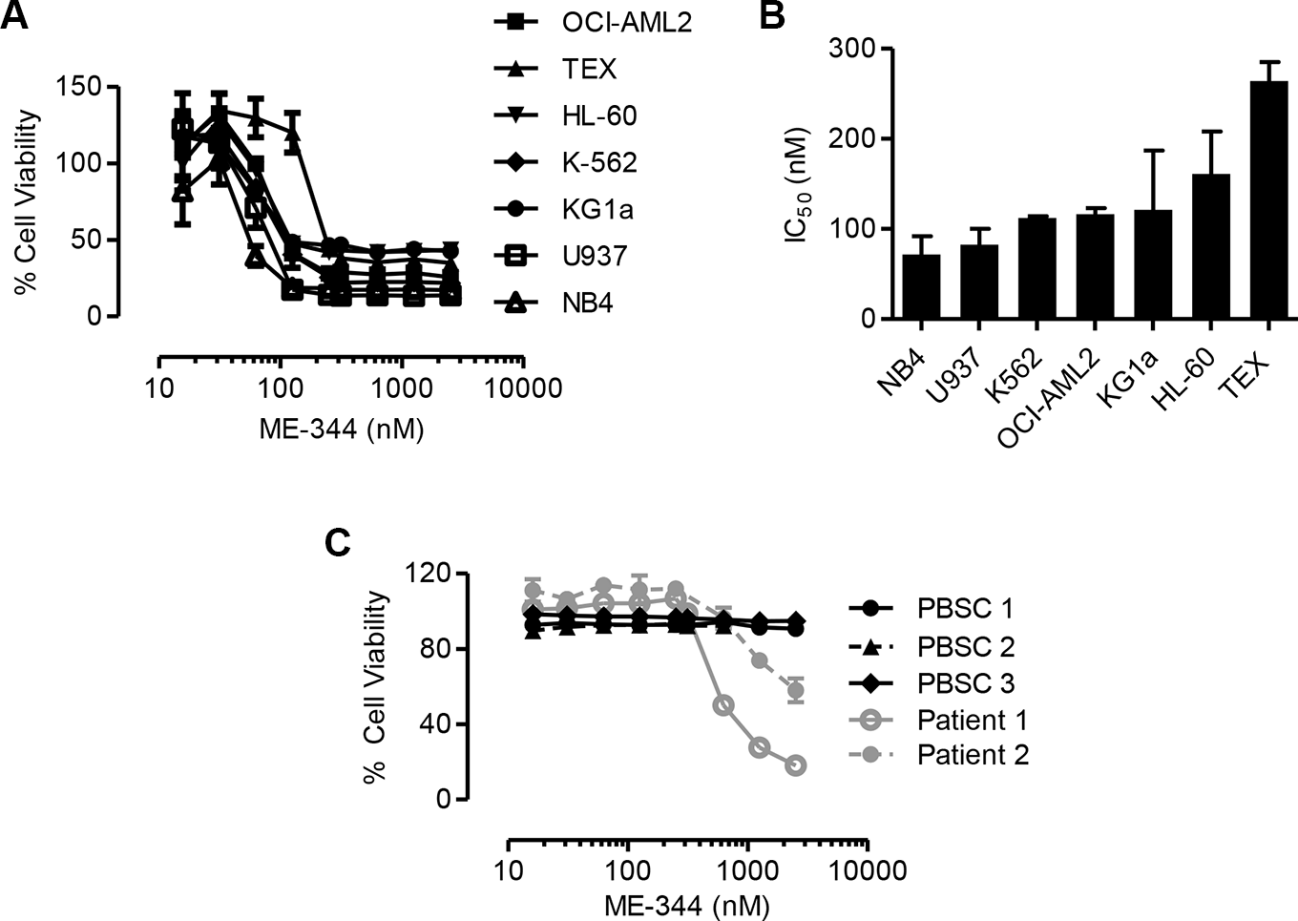
ME-344 is cytotoxic to leukemic cells *in vitro* (**A**) Percentage of viable cells of indicated leukemic cell lines that were treated with increasing concentrations of ME-344. Cell viability was measured by MTS assay after 72 hours of treatment with ME-344. (**B**) IC_50_ values of ME-344 for the indicated leukemic cell lines tested from panel (A). (**C**) Normal hematopoietic samples (black) and primary patient samples (gray) were treated with increasing concentrations of ME-344. After 72 hours of treatment, cell viability was measured by Annexin V and PI staining followed by flow cytometry. Each line represents an independent donor. Data represent mean ± SD of three independent experiments. The lines are connecting the data points. IC_50_ values were calculated using the Spline/LOWESS method in GraphPad Prism.

To determine the potential of ME-344 in hematological malignancies, we evaluated the cytotoxicity of ME-344 against a panel of leukemic cell lines. Cells were treated with increasing concentrations of ME-344 to determine IC_50_ values. ME-344 reduced cell growth and viability of the tested cell lines with IC_50_ values in the range of 70–260 nM (Figure 1A and 1B). We confirmed these results using the SRB assay to measure growth and viability after ME-344 treatment in OCI-AML2, TEX and HL-60 cell lines (Supplementary Figure 1). To further establish the anti-leukemic potential of ME-344, we treated primary AML (gray lines) and normal hematopoietic (black lines) cells with increasing concentrations of ME-344. While ME-344 was toxic to AML patient samples with an IC_50_ of 630 nM and 4 μM, normal hematopoietic cells remained viable in the concentrations tested (Figure 1C).

### ME-344 displays anti-tumor activity in a leukemia xenograft model

**Figure 2:**
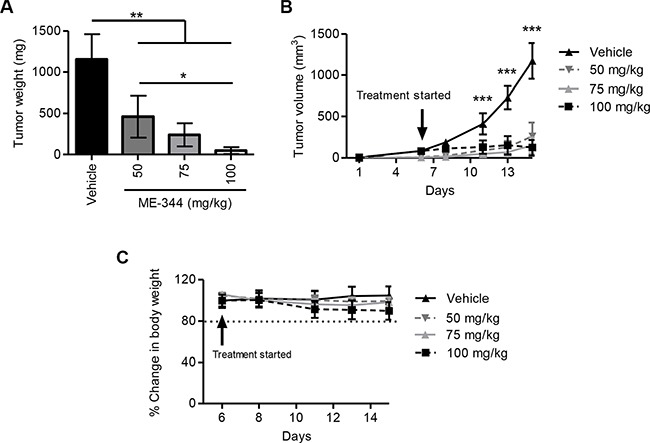
ME-344 displays antitumor activity in an *in vivo* xenograft mouse model Tumor derived OCI-AML2 human leukemia cells (1 × 10^6^) were injected subcutaneously into the flanks of male SCID mice. When tumors were palpable, animals were separated into four groups and were treated with ME-344 by i.p. injection (50, 75, and 100 mg/kg every other day) or vehicle control (*n* = 8 per group). Tumor weight (**A**), tumor volume (**B**), and body weight (**C**) were monitored over time. Mean ± SD; the lines are connecting the data points. *P < 0.01, **P < 0.001, and ***P < 0.0001 from a one-way ANOVA in panel (A) and a two-way ANOVA with Bonferroni posttests comparing all treatment groups in panel (B).

To establish the anti-leukemic effects of ME-344 in *vivo*, we used a leukemia xenograft model and injected OCI-AML2 cells subcutaneously into SCID mice. Once tumors were palpable, mice were treated with intraperitoneal injections of ME-344 (50, 75, and 100 mg/kg) or vehicle control every other day. Compared to vehicle control, ME-344 significantly reduced tumor growth (**P < 0.001 and ***P < 0.0001) (Figure 2A and 2B) without any evidence of toxicity as measured by changes in weight (Figure 2C). In comparison between doses, a significant decrease in tumor weight was observed at 100 mg/kg compared to 50 mg/kg (*P < 0.01). In addition, no changes in behavior as well as gross and histologic appearance of organs at necropsy were observed (Supplementary Figure 2). We reconfirmed these results by using another cell line MDAY-D2 (murine lymphosarcoma) in this leukemia xenograft model (Supplementary Figure 3). Thus, ME-344 displays pre-clinical efficacy against leukemia *in vitro* and *in vivo*.

### Mitochondrial ROS generated by ME-344 does not contribute to cell death

**Figure 3:**
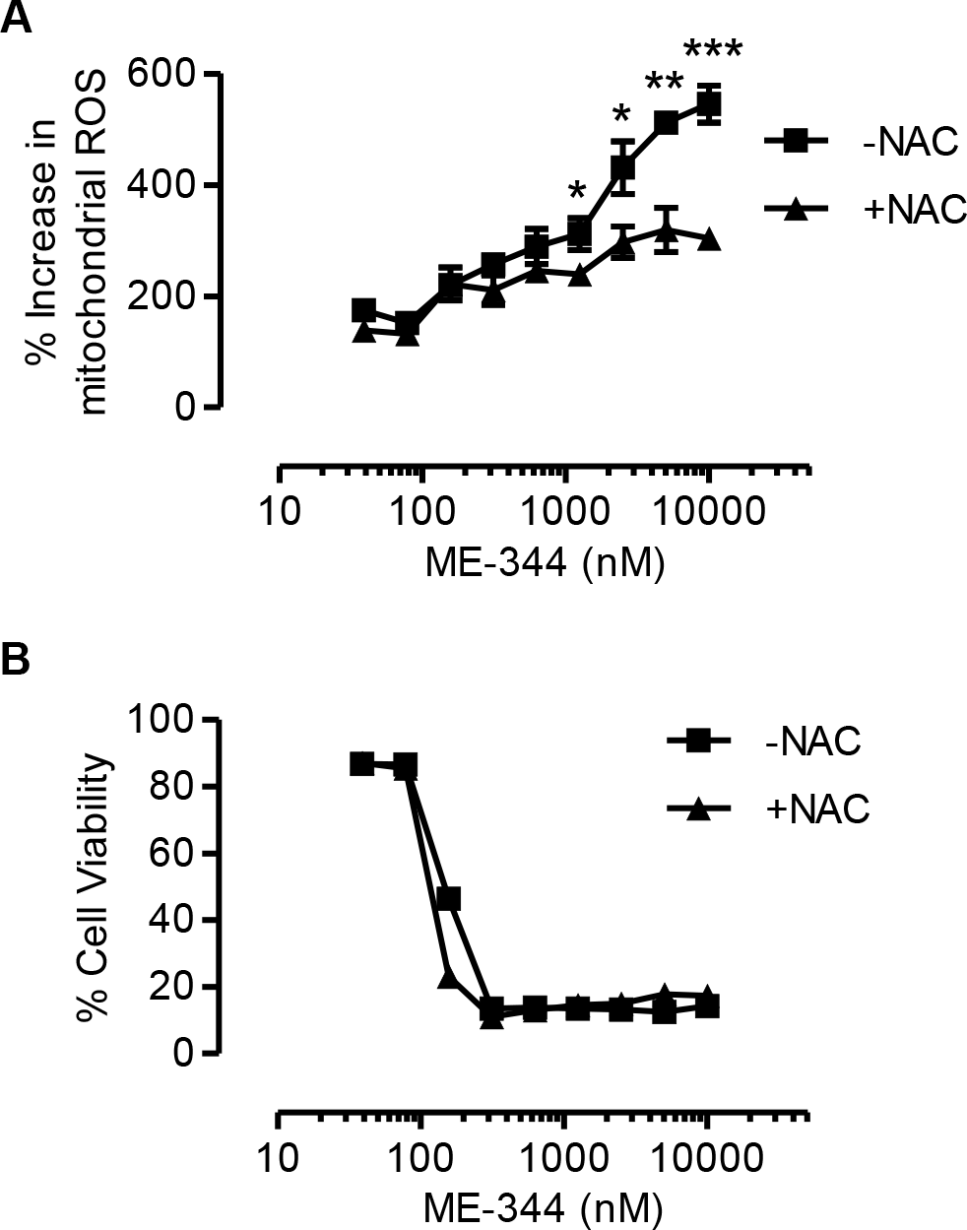
Antioxidant treatment does not rescue cell death induced by ME-344 OCI-AML2 cells were treated with increasing concentrations of ME-344 alone, and ME-344 with 5 mM NAC simultaneously for 72 hours. Accumulation of mitochondrial ROS was assessed by flow cytometry using Mitosox staining (**A**), and cell viability was measured by 7-AAD staining (**B**). Data represent mean ± SD of three independent experiments. The lines are connecting the data points. *P < 0.05, **P < 0.01 and ***P < 0.001 from Student's *t*-test by comparing the means at a given concentration of ME-344.

It has been demonstrated that ME-344 induced death in solid tumor cells by increasing mitochondrial ROS levels and disrupting the mitochondria [9]. Therefore, we investigated mitochondrial ROS generation in leukemic cells after treatment with ME-344. Similar to the previous study, treatment of ME-344 increased mitochondrial ROS production in OCI-AML2 cells (Figure 3A). To test if mitochondrial ROS was responsible for ME-344-induced cell death, we co-treated OCI-AML2 cells with ME-344 and N-acetylcysteine (NAC), an antioxidant that replenishes the GSH-GSSG system [14–17]. While co-treatment with NAC reduced levels of mitochondrial ROS (Figure 3A), it failed to rescue cell death induced by ME-344 in OCI-AML2 cells (Figure 3B). Thus, our results demonstrate that at least in leukemia cells, ME-344-mediated cell death is independent of mitochondrial ROS.

### ME-344 exerts anti-leukemic activity by targeting tubulin

Treatment of cells with ME-344 induced changes in cellular morphology with perturbations in the shape of the plasma membrane. Similar changes in morphology were noted when treating cells with the tubulin inhibitor vinblastine (Supplementary Figure 4). Thus, we postulated that ME-344 might target the cytoskeleton.

**Figure 4:**
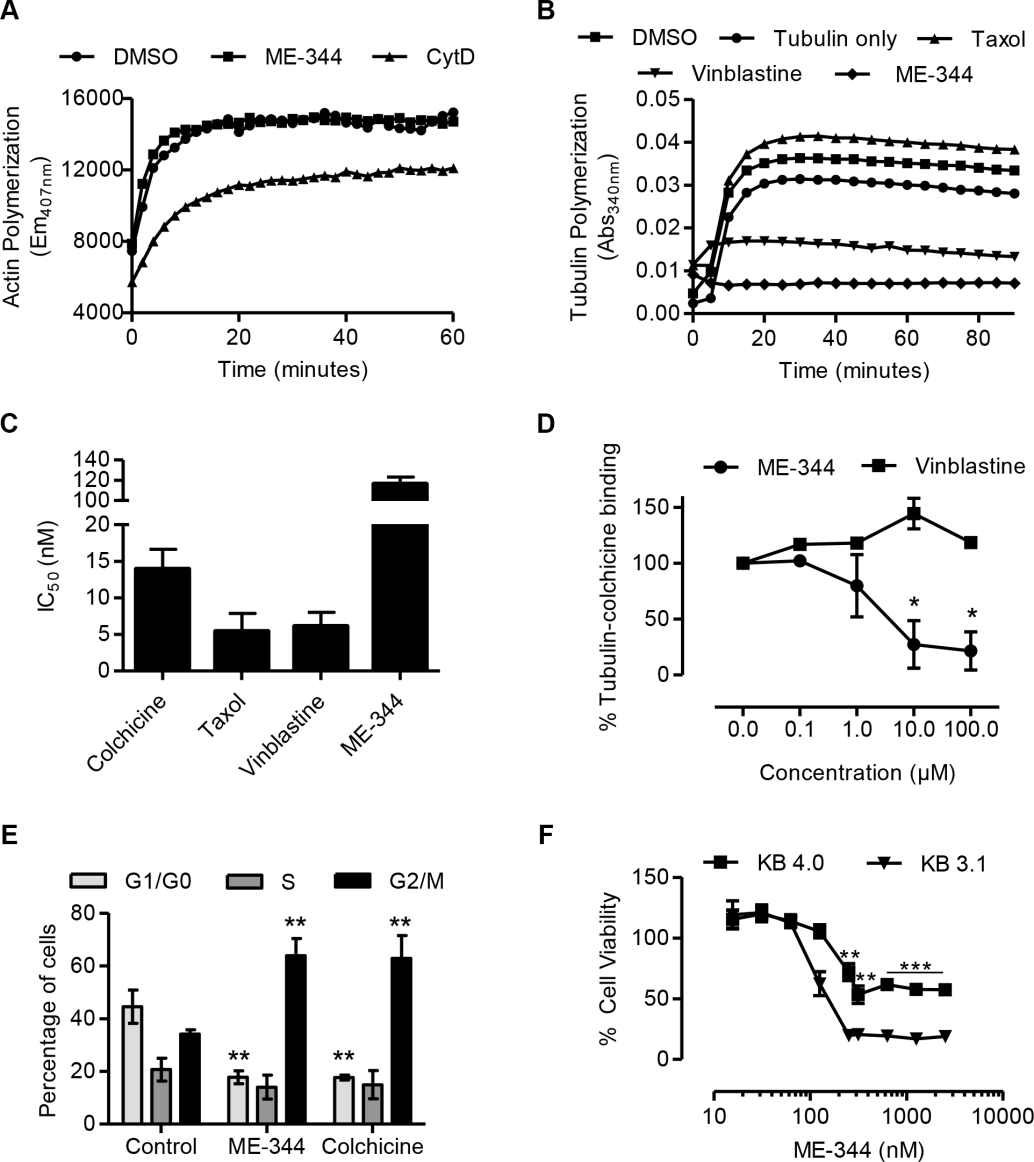
ME-344 targets the colchicine-binding site of tubulin *in vitro* (**A**) Effect of 20 μM ME-344 and 20 μM cytochalasin D (CytD) on actin polymerization as measured by following the increase in fluorescence during conversion of pyrene G-actin (monomer) to pyrene F-actin. (**B**) Effect of 10 μM ME-344, 10 μM taxol, and 10 μM vinblastine on tubulin polymerization, as measured by an increase in absorbance. (**C**) OCI-AML2 cells were treated with increasing concentrations of colchicine, taxol, vinblastine and ME-344. Cell viability was measured by MTS assay after 72 hours of treatment and IC_50_ values were calculated. (**D**) Percent tubulin-colchicine binding after incubating tubulin with increasing concentrations of ME-344 and vinblastine. (**E**) OCI-AML2 cells were treated with 100 nM of ME-344 and colchicine to observe the impact on cell cycle. (**F**) KB-3.1 and KB-4.0-HTI36 cells were treated with increasing concentrations of ME-344. Cell viability was measured by MTS assay after 72 hours of treatment. Panels (A) and (B) show representative results, panels (C) and (F) show mean ± SD of three independent experiments, and panels (D) and (E) show mean ± SD of two independent experiments. *P<0.05, **P < 0.01 and ***P < 0.001 from Student's *t*-test by comparing the means at a given concentration in panels (D) and (F), and from two-way ANOVA in panel (E).

We investigated the effects of ME-344 on actin and tubulin polymerization using *in vitro* assays. While, ME-344 did not have a notable effect on actin polymerization, treatment with the known actin dynamics inhibitor, cytochalasin D, reduced the rate of actin polymerization (Figure 4A). On the other hand, ME-344 completely suppressed tubulin polymerization in the cell-free assay. As expected, taxol (paclitaxel), a known promoter of tubulin polymerization, enhanced tubulin polymerization, whereas vinblastine, a known inhibitor of tubulin polymerization, suppressed tubulin polymerization (Figure 4B). In OCI-AML2 cells, similar to colchicine, taxol, and vinblastine, ME-344 caused cell death with IC_50_ values in nM (Figure 4C).

To determine the interaction site of ME-344 with tubulin, we used a competitive colchicine-binding assay that is based on the fluorescent properties of the colchicine-tubulin complex [18]. Our results show that ME-344 was able to compete with colchicine indicating that ME-344 targets tubulin by interacting at the colchicine binding site. This site is distinct from where vinblastine binds tubulin, since vinblastine did not compete with colchicine (Figure 4D). To further confirm that ME-344 targets tubulin polymerization, we treated cells with ME-344 and studied the impact on cell cycle. Similar to the effect of known tubulin inhibitors [19], ME-344 caused G2/M specific cell cycle arrest due to disrupting the microtubule assembly (Figure 4E).

### Tubulin resistant cell lines have decreased sensitivity to ME-344

Having established that ME-344 targets tubulin *in vitro*, we investigated if inhibiting tubulin polymerization was functionally important for the toxicity of ME-344. We used the paired epidermoid carcinoma cell lines KB-3.1, with wild type tubulin, and KB-4.0-HTI36 a cell line resistant to tubulin inhibitors due to a single nucleotide substitution in α-tubulin [20]. Consistent with our *in vitro* observations, KB-4.0-HTI36 cells had at least a 3-fold higher IC_50_ value and displayed resistance to ME-344 (Figure 4F) similar to their resistance to colchicine and vinblastine (Supplementary Figure 5). On the other hand, both cell lines had similar IC_50_ values for taxol that promotes tubulin polymerization (Supplementary Figure 5) which is consistent with the original observation by the group that developed these cell lines [20].

To further explore the effects of ME-344 on tubulin polymerization, we visualized the microtubule assembly using immunofluorescence in PPC-1 cells following treatment with colchicine and ME-344. We found that ME-344 causes morphological changes in the tubulin assembly (Supplementary Figure 6). Thus, inhibition of tubulin by ME-344 is functionally important for its cytotoxicity.

### ME-344 synergizes with vinblastine and is additive with AraC in leukemic cells

**Figure 5:**
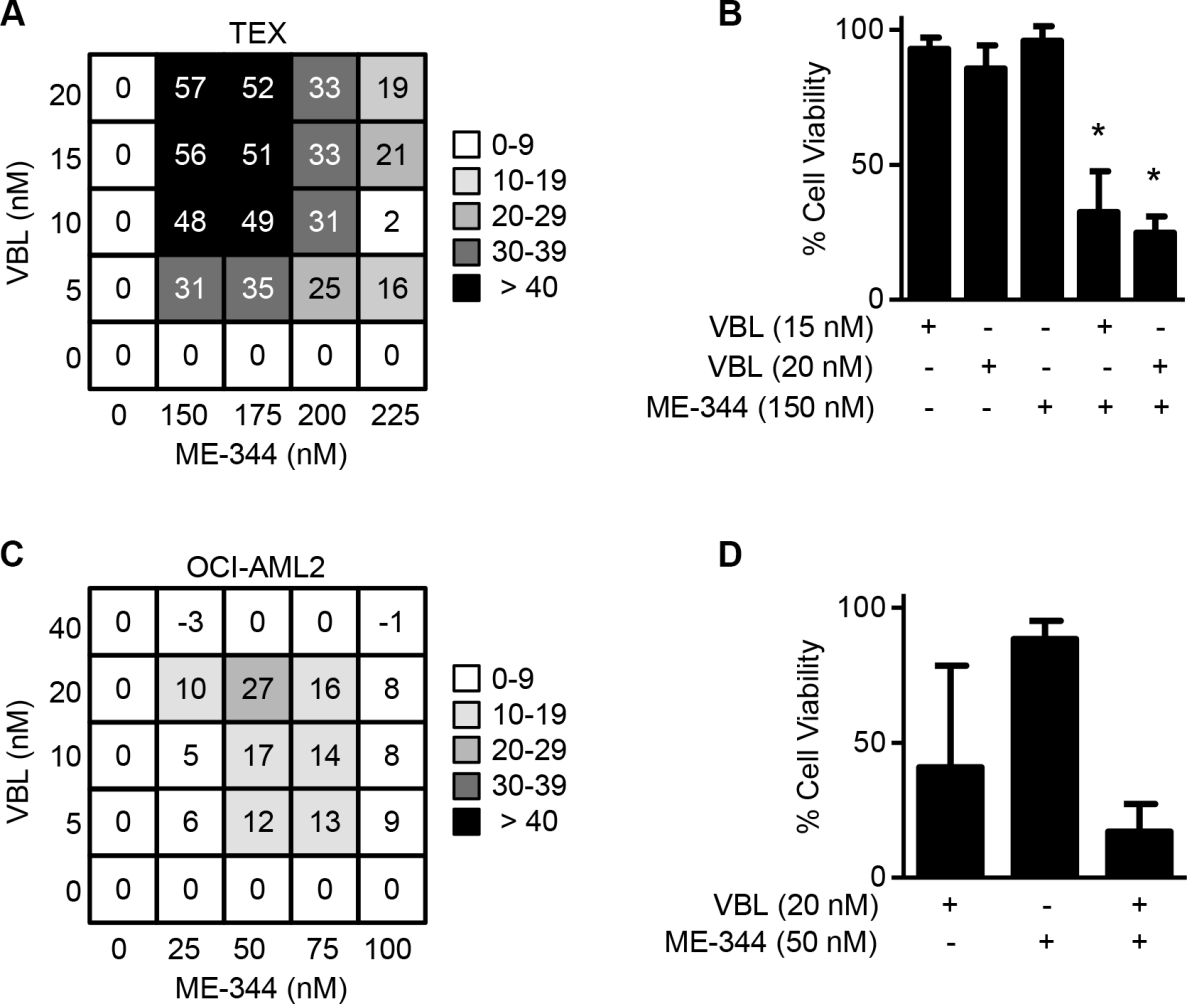
ME-344 synergizes with vinblastine in TEX cells and OCI-AML2 cells (**A** and **B**) TEX and (**C** and **D**) OCI-AML2 cells were treated with the indicated concentrations of ME-344 and vinblastine (VBL) simultaneously. Cell viability was assessed using the MTS assay after 72 hours. Excess Over Bliss values above 10 were assigned as being synergistic. Representative synergistic combinations in TEX (B) and in OCI-AML2 (D) are shown. *P < 0.0001 from one-way ANOVA. Data represent mean ± SD of three independent experiments.

Since tubulin inhibitors bind to many diverse sites on tubulin, the combination of two or more of these inhibitors can improve their efficacy [19]. Therefore, to evaluate the extent of synergy of ME-344 with other tubulin inhibitors and anticancer drugs, we carried out treatments of different drug combinations in TEX and OCI-AML2 leukemia cell lines. Cells were treated with increasing concentrations of ME-344 along with increasing concentrations of vinblastine, cytarabine, daunorubicin, and the kinase inhibitors imatinib, sunitinib, and quizartinib. Cell growth and viability was measured 72 hours after incubation by MTS assay. Synergy, additivism, and antagonism were assessed using Excess Over Bliss Additivism [21, 22]. ME-344 showed strong synergy with vinblastine to reduce the growth and viability of TEX cells and showed moderate synergy in OCI-AML2 cells (Figure 5A–5D) (*P < 0.0001). In addition, ME-344 showed synergy with cytarabine to reduce the growth of both TEX and OCI-AML2 cells (Supplementary Figure 7). The combinations with the other agents were primarily additive or antagonistic at high drug concentrations (Supplementary Figures 8 and 9).

## DISCUSSION

In this study, we demonstrated that ME-344 displays potent anti-leukemic activity using *in vitro* and *in vivo* methods. Thus, these data, along with the prior clinical efficacy of ME-344, support the clinical evaluation of ME-344 for patients with acute leukemia. In a previous phase I dose escalation clinical trial of ME-344 in patients with solid tumors, a maximal tolerated dose (MTD) of 10 mg/kg was established. Notably, in this clinical trial, a partial response for ≥ 52 weeks was reported in one patient with small cell lung cancer, and four patients had prolonged stable disease [12].

To date, the mechanism of action and molecular targets of ME-344 have not been fully defined. Similar to previous studies, we demonstrated that ME-344 promoted mitochondrial ROS generation. However, increased mitochondrial ROS did not completely explain the mechanism of action of ME-344 in leukemia, suggesting that ME-344 may have other cellular targets. Therefore, we used *in vitro* assays to identify the cellular target of ME-344 in leukemia cells. We confirmed that ME-344 inhibited tubulin polymerization and interacted with tubulin at the colchicine binding site. This site is distinct from the binding site of vinca alkaloids. Thus microtubules appear to be at least one important molecular target for ME-344. In support of our results, another isoflavone glaziovianin A displayed a pattern of differential cytotoxicity in a panel of 39 cancer cell lines that suggested that it induced cell death by inhibiting tubulin polymerization [23]. In addition, it is noteworthy that neuropathy, which is a common toxicity of tubulin inhibitors, was a dose limiting toxicity in the phase I trial of ME-344 [12].

Further studies will be necessary to understand how inhibition of tubulin polymerization by ME-344 may be related to increased ROS generation. Potentially, these two findings could be linked through alterations in mitochondrial motility. By altering tubulin polymerization, ME-344 could impact mitochondrial motility leading to mitochondrial dysfunction. This possibility is supported by findings that 1-methyl-4-phenylpiridinium (MPP(+)), the parkinsonian toxin, alters microtubule dynamics resulting in mitochondrial transport impairment and cell death [24].

In conclusion, ME-344 is a novel isoflavone based tubulin inhibitor that binds tubulin at a site distinct from vinca alkaloids. It has effective anti-leukemic properties *in vivo* and *in vitro*. Taken together with data from clinical trials in solid tumors, our study supports a clinical trial of ME-344 in patients with AML and other hematological malignancies.

## MATERIALS AND METHODS

### Cell culture

All leukemic cell lines were grown in IMDM media supplemented with 10% fetal bovine serum (FBS), 100 μg/ml penicillin and 100 U/ml streptomycin (all from Hyclone, Logan, UT). TEX cells were grown in the presence of 15% FBS, 100 μg/ml penicillin, 100 U/ml streptomycin, 2 mM L-glutamine, 20 ng/ml SCF, and 2 ng/ml IL-3. The epidermoid carcinoma cell lines KB-3.1 and KB-4.0-HTI36 (a gift from Dr. F. Loganzo, Pearl River, NY) were grown in Dulbecco's Modified Eagle Medium (DMEM) supplemented with 10% FBS. All cells were incubated at 37°C in a humidified air atmosphere supplemented with 5% CO_2_. Primary human AML samples were isolated from peripheral blood from consenting patients with AML, who had at least 80% malignant cells among low-density cells. AML cells were isolated by Ficoll density centrifugation. Except where otherwise noted, primary normal hematopoietic cells refer to normal mononuclear cells obtained from healthy consenting volunteers donating peripheral blood stem cells (PBSCs) for allogeneic stem cell transplantation after G-CSF mobilization. Primary cells were cultured at 37°C in IMDM, supplemented with 20% FBS, 100 μg/ml penicillin and 100 U/ml streptomycin. The University Health Network institutional review board approved the collection and use of human tissue for this study.

### Cell growth and viability assays

The MTS assay was used for assessment of cell growth and viability as per the manufacturer's instructions (Promega, Madison, WI). Cell death was measured by Annexin V-fluoroscein isothiocyanate (FITC) and Propidium Iodide (PI) staining (Biovision Research Products, Mountain View, CA) or by 7-AAD staining (BD biosciences) using flow cytometry (FACS canto II, Becton Dickinson, FL) according to the manufacturer's instructions. Results were analyzed with FlowJo (TreeStar, Ashland, OR). IC_50_ values were calculated using the Spline/LOWESS method in GraphPad Prism.

### Tubulin polymerization and binding assays

MAP-rich bovine tubulin (Cytoskeleton, Denver, CO) was reconstituted in ice-cold polymerization buffer (80 mM PIPES pH 6.9, 0.5 mM EGTA, 2 mM MgCl_2_, 10% glycerol and 1 mM GTP) at a concentration of 1.2 mg/ml and centrifuged at top speed for 5 min at 4°C. Supernatant (100 μl/well) was added to ME-344 or the appropriate controls in a 96-well plate to obtain a final drug concentration of 10 μM. Absorbance was measured at 340 nm every 5 min for 90 min at 37°C.

Competitive inhibition of colchicine binding to tubulin was performed using purified porcine tubulin (Cytoskeleton, Denver, CO) reconstituted at 0.5 mg/ml (80 mM PIPES pH 6.9, 0.5 mM EGTA and 2 mM MgCl_2_). Briefly, the tubulin solution (300 μl/sample) was incubated at 37°C for 60 min in the presence of compounds at varying concentrations and colchicine (12 μM). Fluorescence of the colchicine-tubulin complex was measured in disposable cuvettes using excitation and emission wavelengths of 360 nm and 430 nm, respectively.

### Actin polymerization assays

Actin polymerization was measured using the actin polymerization Biochem kit (Cytoskeleton, Denver, CO). Briefly, 1 mg of pyrene labeled muscle actin was resuspended with 50 μl of ice cold sterile de-ionized water and was made up to a final concentration of 0.4 mg/ml using 5 mM Tris-HCl pH 8.0, 0.2 mM CaCl_2_ and 200 mM ATP. The assay was performed in a 96-well plate with the appropriate controls. The samples were excited at 365 nm and the emission was collected at 407 nm. The plate was placed in the fluorimeter and samples were read once every 60 sec for a total of 3 min to establish a baseline fluorescent measurement for all samples. After 3 min, 20 μl of ME-344 at a final concentration of 20 μM was added and the plate was read for a further 20 min to test if ME-344 induces polymerization. After 20 min, 20 μl of 10× Actin Polymerization Buffer was added and plate was read for 1 hour every 20 sec or until the fluorescent signal plateaued.

### Cell cycle analysis

OCI-AML2 cells were treated with 100 nM of ME-344 or colchicine overnight, harvested, washed once in cold PBS and fixed overnight in cold 70% ethanol at −20°C. Cells were then pelleted, washed once with PBS and treated with 100 ng/ml DNase-free RNase A (Invitrogen, Carlsbad, CA) at 37°C for 30 minutes. Propidium Iodine (5 μg/ml) solution was added prior to measuring DNA content on a FACScalibur (Becton Dickinson, FL). Results were analyzed with FlowJo (TreeStar, Ashland, OR).

### Measurement of mitochondrial ROS

Mitochondrial reactive oxygen species (ROS) was detected by staining cells with MitoSOX (5 μM) and followed by flow cytometric analysis as described previously [25, 26]. Briefly, cells were stained with MitoSOX in HBSS buffer at 37°C for 30 min, and then re-suspended in binding buffer with a viability dye (Annexin V or 7-AAD to identify viable cells and assess their reactive oxygen intermediate levels. Data were analyzed with FlowJo version 7.7.1 (TreeStar).

### Assessment of the anti-leukemia activity of ME-344 in mouse models of human leukemia

OCI-AML-2 human leukemia cells (1 × 10^6^) were injected subcutaneously into the flanks of male SCID mice (Ontario Cancer Institute, Toronto, ON). When the tumors were palpable, mice were treated with ME-344 by i.p. injection (50, 75, or 100 mg/kg once every other day) over 11 days in saline or vehicle control (*n* = 8 per group). Caliper measurements were used to calculate tumor volumes (volume = tumor length × width^2^ × 0.5236) over time. At the end of the experiment, mice were sacrificed and the tumors excised and weighed.

## SUPPLEMENTARY MATERIALS FIGURES


