# Supplementary material for: A novel isoflavone, ME-344, targets the cytoskeleton in acute myeloid leukemia
Source: Oncotarget. 2016 Jul 6;7(31):49777–85. doi: 10.18632/oncotarget.10446 (PMC5226547; doi:10.18632/oncotarget.10446)
Supplement: Supplementary file 1 [file oncotarget-07-49777-s001.pdf]

# A novel isoflavone, ME-344, targets the cytoskeleton in acute myeloid leukemia

## Supplementary Materials

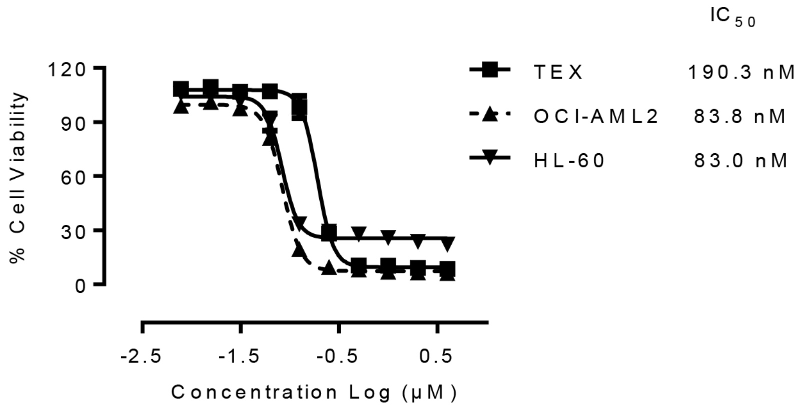


**Supplementary Figure S1: ME-344 is cytotoxic to leukemic cell *in vitro*.** OCI-AML2, HL-60 and TEX leukemic cell lines were treated with increasing concentrations of ME-344. After 72 hours of treatment, cell viability was measured by SRB assay. Data represent Mean ± SEM.

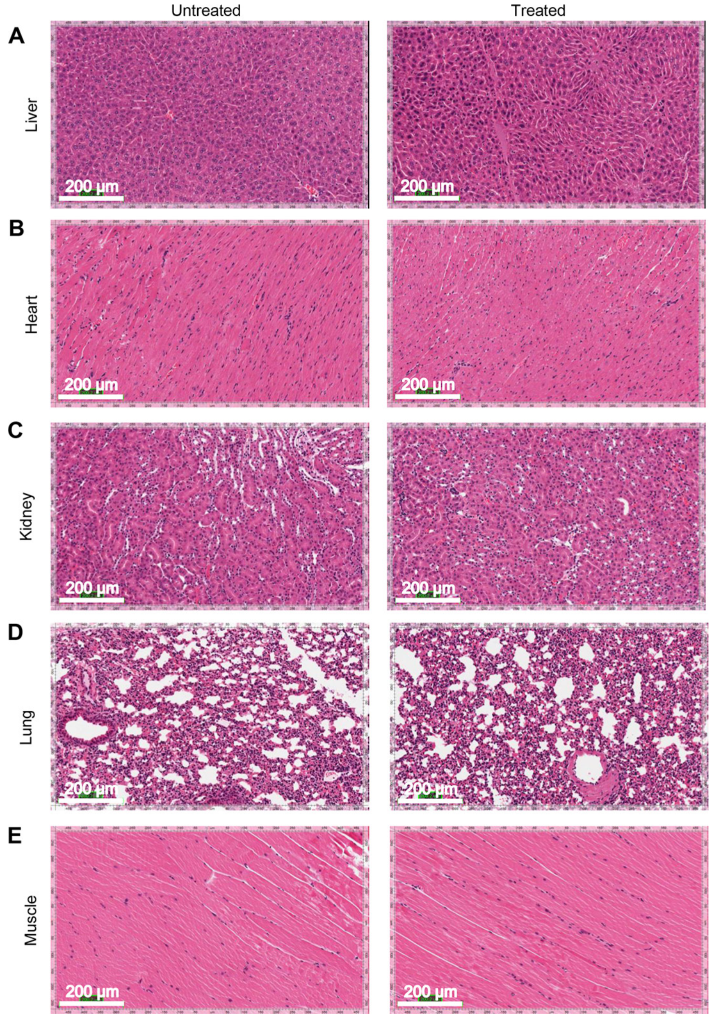

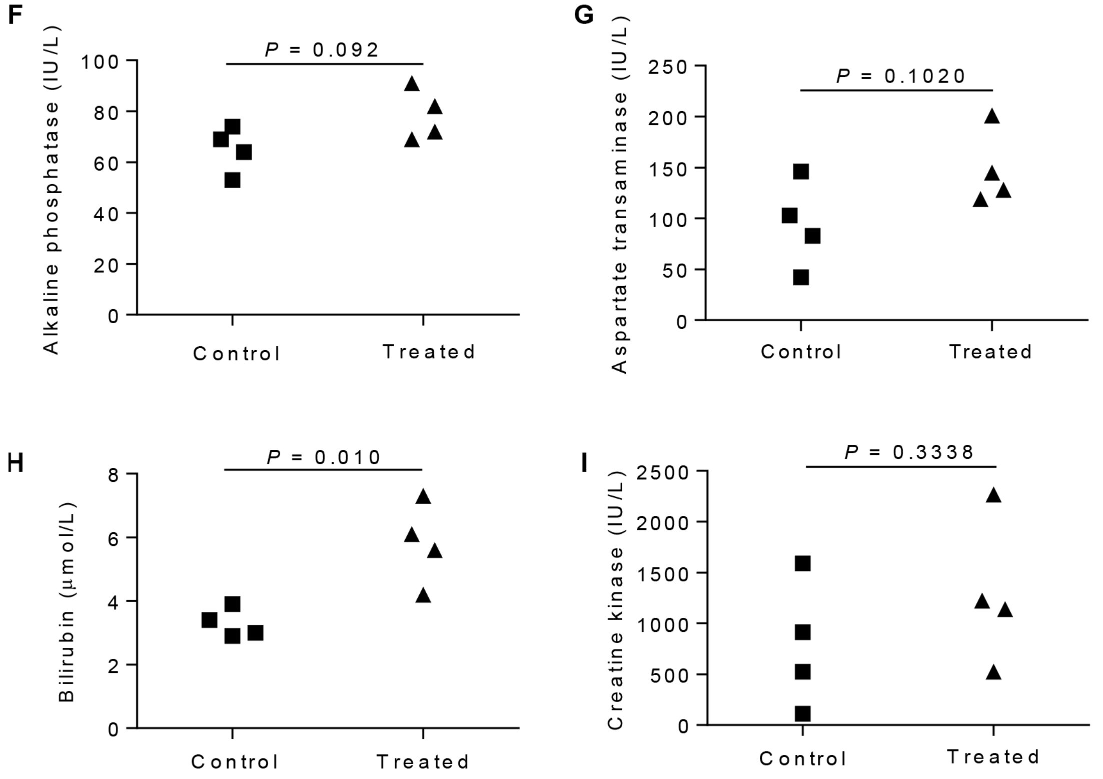


**Supplementary Figure S2: Toxicology of ME-344 treatment studies *in vivo*.** (**A**–**E**) Gross organ histology of SCID mice treated with ME-344 (100 mg/kg every other day by i.p injection). (A) Liver, (B) Heart, (C) Kidney, (D) Lung, (E) Muscle sections stained with hematoxylin and eosin are shown (10× magnification). Representative sections from the tissues are shown. Scale bars: 200 μm. Renal and liver function was determined in mice treated using (**F**) alkaline phosphatase, (**G**) aspartate transaminase, (**H**) bilirubin, and (**I**) creatine kinase measurements. *P* values were calculated using two-tailed unpaired Student's *t*-test.

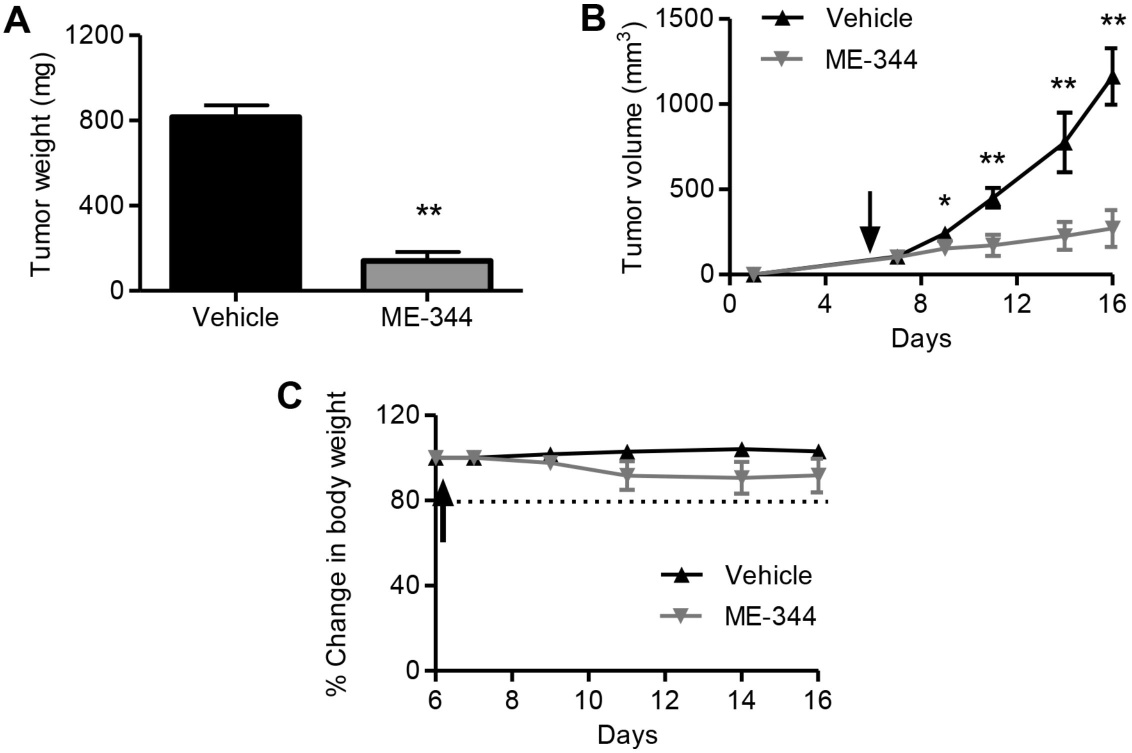


**Supplementary Figure S3: ME-344 displays antitumor activity in an *in vivo* xenograft mouse model.** MDAY murine lymphosarcoma cells were injected subcutaneously into the flanks of male SCID mice. When tumors were palpable, animals were separated into two groups and were treated with ME-344 by i.p. injection (100 mg/kg every other day) or vehicle control ($n$ = 7 per group). Tumor weight (**A**), tumor volume (**B**), and body weight (**C**) were monitored over time. Arrow indicates the beginning of treatment on day 6. Mean ± SD; the lines are connecting the data points. *$P < 0.01$ and **$P < 0.0001$ from unpaired Student's *t*-test comparing the mean at each day.

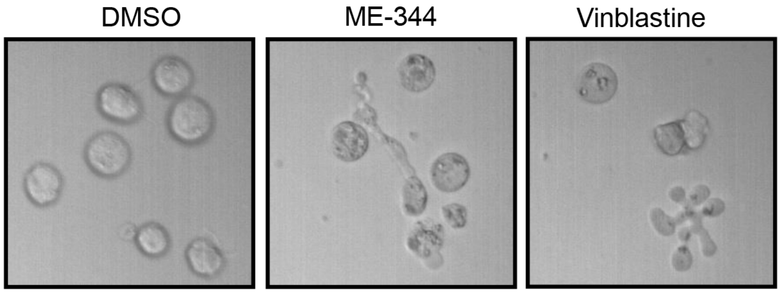


**Supplementary Figure S4: ME-344 causes cellular morphological changes similar to vinblastine.** OCI-AML2 cells were treated for 24 hours with 100 nM ME-344 or 10 nM vinblastine and images were acquired with a Zeiss Axio Observer at 32× magnification. Scale bar = 10 µm.

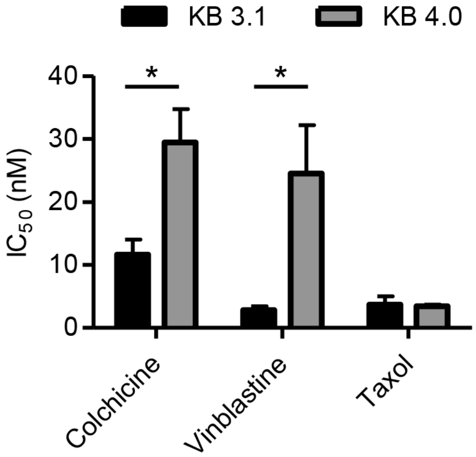


**Supplementary Figure S5: KB-4.0-HTI36 cells are resistant to colchicine and vinblastine, but not to taxol.** KB-3.1 and KB-4.0-HTI36 cells were treated with increasing concentrations of colchicine, vinblastine and taxol. Cell viability was measured by MTT assay after 72 hours of treatment. $IC_{50}$ values were calculated using the Spline/LOWESS method in GraphPad Prism. **P* < 0.01 from unpaired Student's *t*-test.

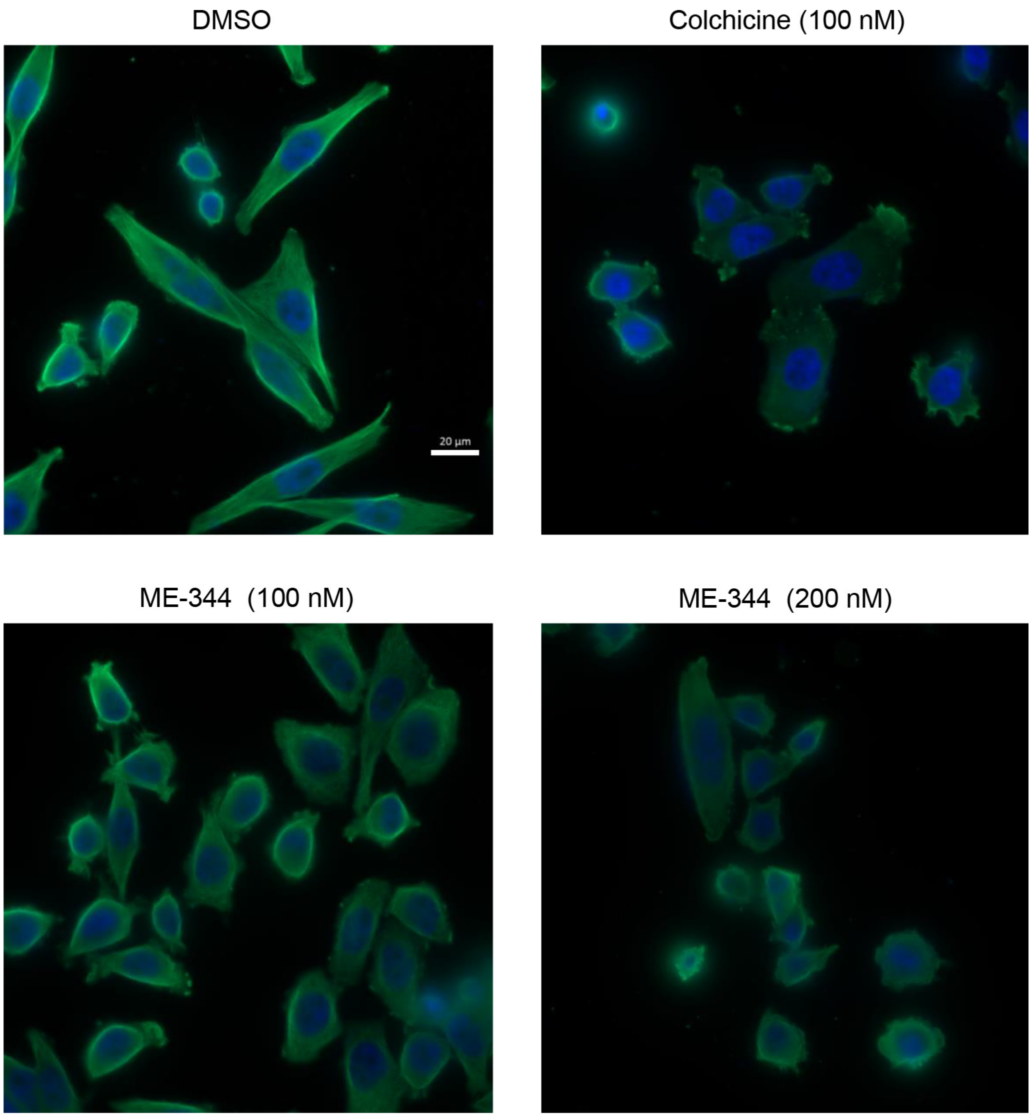


**Supplementary Figure S6: ME-344 treatment in solid tumor cell line.** PPC-1 cells were treated with ME-344, colchicine or DMSO. Tubulin was visualized using an anti-alpha-tubulin antibody and nuclei were stained with DAPI. Scale bar: 20 µm.

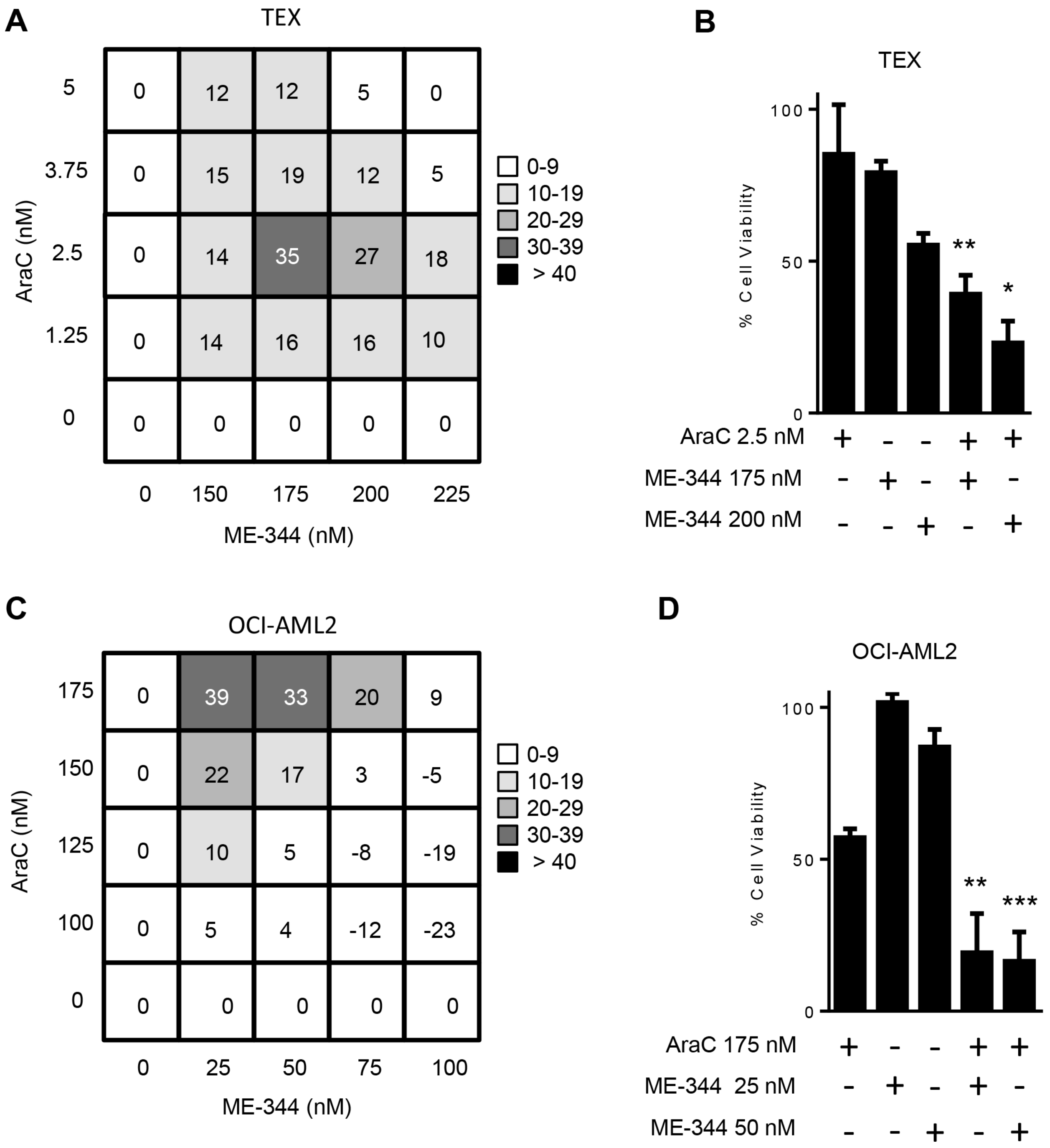


**Supplementary Figure S7: In combination studies, ME-344 is synergistic with Cytarabine (AraC) in the leukemic stem cell line TEX and OCI-AML2 cells.** (**A**) TEX and (**C**) OCI-AML2 cells were treated simultaneously with the indicated concentrations of ME-344 and Ara C. After 72 hours, viability was assessed by MTS assay in the various combinations of concentrations as indicated in the matrix. Representative synergistic combinations in TEX (**B**) and OCI-AML2 (**D**) are shown. *$P < 0.05$, **$P < 0.01$, and ***$P < 0.001$ from one-way ANOVA with Bonferroni post test analysis. Significance shown for combination treatments vs. respective ME-344 individual treatment in panel (B), and combination treatments vs. AraC individual treatment in panel (D). Results represent average of three independent experiments performed in triplicate. Error bars represent standard deviation.

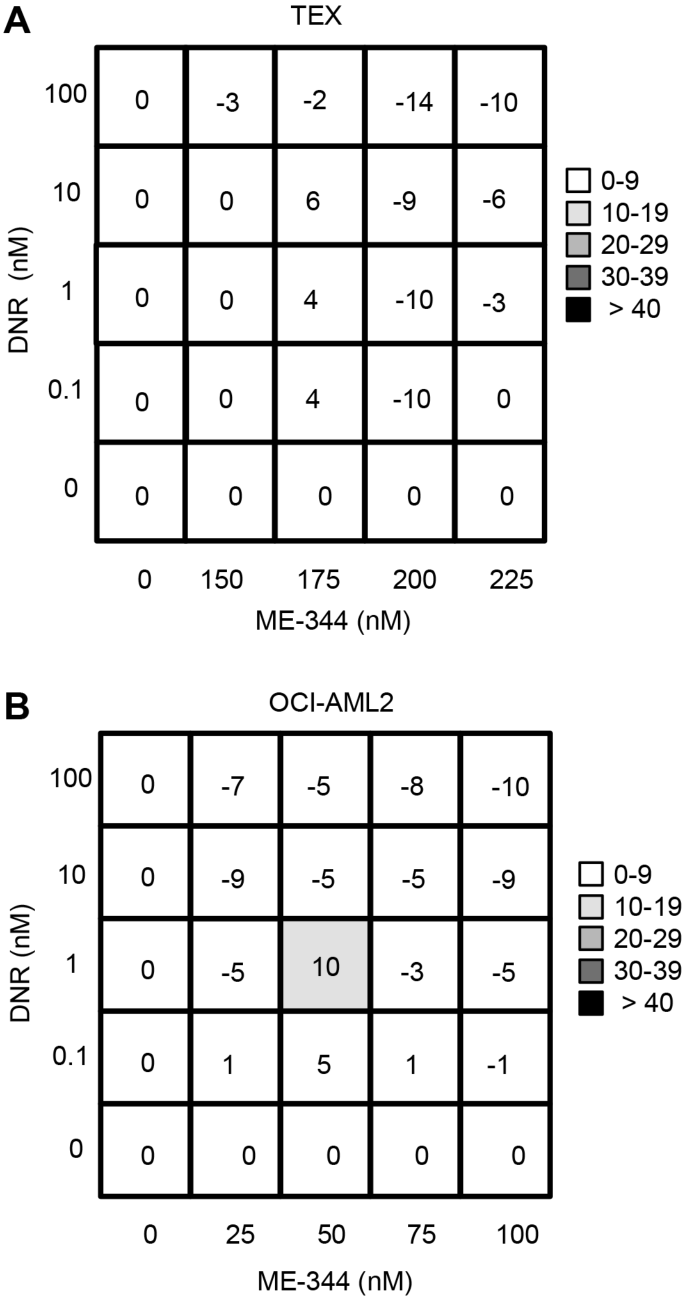


**Supplementary Figure S8: In combination studies, ME-344 is antagonistic with daunorubicin (DNR) in the leukemic stem cell line TEX and OCI-AML2 cells.** (**A**) TEX and (**B**) OCI-AML2 cells were treated simultaneously with the indicated concentrations of ME-344 and DNR. After 72 hours, viability was assessed by MTS assay in the various combinations of concentrations as indicated in the matrix. Results represent average of three independent experiments performed in triplicate.

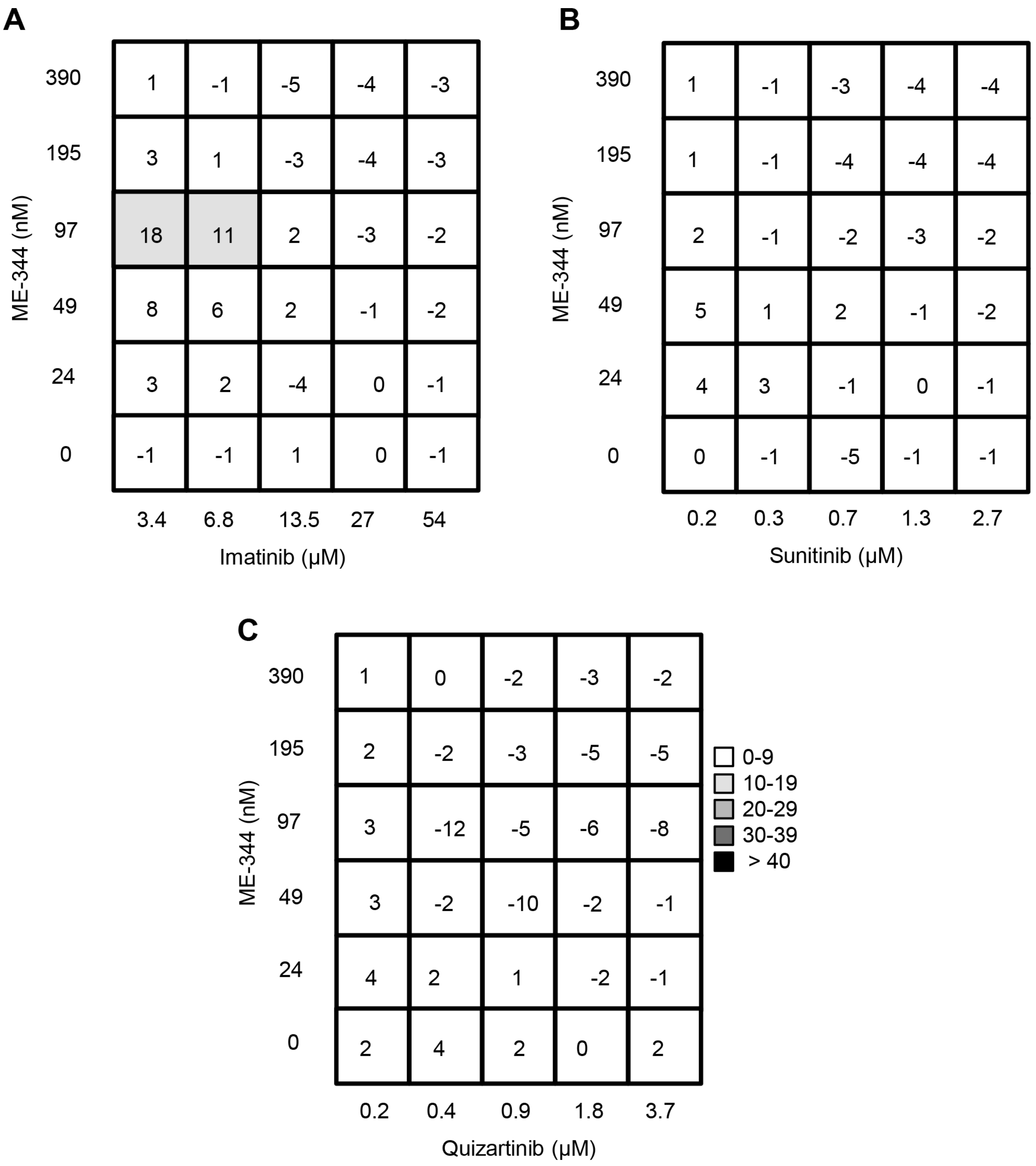


**Supplementary Figure S9: In combination studies, ME-344 is not synergistic with tyrosine kinase inhibitors (TKI) in the leukemic cell line OCI-AML2.** Cells were treated in combination with ME-344 and (**A**) Imatinib (**B**) Sunitinib and (**C**) Quizartinib. After 72 hours, viability was assessed by SRB assay in the various combinations of concentrations as indicated in the matrix. Results represent average of three independent experiments performed in triplicate.
